# Sequencing and Annotation of the Genome of Mycobacterium tuberculosis MYC004, a Strain Causing Meningitis in Mexico

**DOI:** 10.1128/genomeA.00523-18

**Published:** 2018-06-21

**Authors:** Ana Laura Guillén-Nepita, Andrea Monserrat Negrete-Paz, Gerardo Vázquez-Marrufo, Andrés Cruz-Hernández, Pablo Fresia, Hugo Naya, Ma Soledad Vázquez-Garcidueñas

**Affiliations:** a División de Estudios de Posgrado, Facultad de Ciencias Médicas y Biológicas Dr. Ignacio Chávez, Universidad Michoacana de San Nicolás de Hidalgo, Morelia, Michoacán, Mexico; b Facultad de Ingeniería, Universidad Autónoma de Querétaro, Centro Universitario, Santiago de Querétaro, Mexico; c Centro Multidisciplinario de Estudios en Biotecnología, Facultad de Medicina Veterinaria y Zootecnia, Universidad Michoacana de San Nicolás de Hidalgo, Morelia, Michoacán, Mexico; d Escuela de Agronomía, Universidad De La Salle Bajío, León, Guanajuato, Mexico; e Unidad de Bioinformática, Institut Pasteur de Montevideo, Montevideo, Uruguay

## Abstract

Mycobacterium tuberculosis strain MYC004 was isolated from a Mexican patient with tuberculous meningitis, the most aggressive form of tuberculosis. The draft genome sequence is the first of a meningeal strain of M. tuberculosis reported from Latin America and consists of 4,411,530 bp, including 4,251 protein-encoding genes.

## GENOME ANNOUNCEMENT

It is estimated that one-third of the world’s population is infected with Mycobacterium tuberculosis, although the majority will never develop an active disease. There are many types of extrapulmonary tuberculosis affecting every organ system in the body, with tuberculous meningitis being the most fatal form, killing approximately one-third of patients despite antituberculosis treatment (1). The majority of the tuberculosis burden and deaths are concentrated in middle-income countries. There are few studies making attempts to relate extrapulmonary forms of the disease to a specific genetic variant of M. tuberculosis on a genome-wide scale (2–4). In order to continue efforts to identify a genetic variant that could impact disease severity and tissue tropism, more genome sequences of extrapulmonary strains from different geographic regions need to be analyzed. However, no meningeal isolates of M. tuberculosis from Latin America were represented in databases or bioinformatics analyses (4). M. tuberculosis strain MYC004 was isolated by the Laboratorio Estatal de Salud Pública, from the cerebrospinal fluid of a male patient diagnosed in 2012 with tuberculous meningitis in Michoacán, Mexico. The strain was cultured at 37°C on Lowenstein-Jensen agar for 8 weeks. Genomic DNA was extracted in accordance with previously described methods (5); it was sequenced using the ABI SOLiD sequencing platform with a 50-bp-long single-end library, and a total of 1,323,759 reads were generated. Raw reads in color space fasta format were trimmed, and a reference-guided assembly was performed using SOLiD BioScope version 1.3 (Applied Biosystems) for mapping the reads to the reference genome M. tuberculosis H37Rv (6). Annotation was performed using Prokka (7) and Rapid Annotations using Subsystems Technology (RAST) (8). The genes were validated using the external gene databases Mycobrowser (https://mycobrowser.epfl.ch/) (9) and UniProtKB (http://www.ebi.ac.uk/uniprot) (10). The annotations were manually reviewed and curated using Artemis (11). The length of the draft genome of M. tuberculosis MYC004 is 4,411,530 bp, with a GC content of 64.7%. In total, 4,251 protein-coding genes were annotated, including 51 RNA-coding genes, 48 tRNAs, and 3 rRNAs. Of the total protein-coding genes, 2,955 (69.51%) were functionally assigned, and the remaining genes were annotated as hypothetical proteins. A total of 386 subsystems were annotated in the M. tuberculosis MYC004 genome using the RAST server, with the main subsystems being amino acids, amino acid derivatives, carbohydrates, cofactors, vitamins, prosthetic groups, and pigments.

A comparison of genome sequences using RAST revealed that the strains most similar to M. tuberculosis MYC004 are M. tuberculosis NCGM2209 (score, 483), M. tuberculosis F11 (score, 427), and M. tuberculosis UM 1072388579 (score, 420).

### Accession number(s).

The draft genome sequence of M. tuberculosis MYC004 was deposited in GenBank under the accession number CP024614.
